# Predictive performance of machine learning models in acute ischemic stroke: a systematic review and meta-analysis

**DOI:** 10.3389/fneur.2026.1771341

**Published:** 2026-03-11

**Authors:** Uzma Khanum, Vasudeva Guddattu, Shasthara Paneyala, Asha Srinivasan, Chaithra Nagaraju

**Affiliations:** 1 Division of Medical Statistics, School of Life Sciences, JSS Academy of Higher Education and Research, Mysuru, India; 2 Department of Applied Statistics and Data Science, Manipal Academy of Higher Education, Udupi, India; 3 Department of Neurology, JSS Academy of Higher Education and Research, Mysuru, India; 4 Division of Nanoscience and Technology, School of Life Sciences, JSS Academy of Higher Education and Research, Mysuru, India

**Keywords:** acute ischemic stroke, machine learning, prognosis, outcome prediction, meta-analysis

## Abstract

**Introduction:**

Acute ischemic stroke (AIS) is a leading cause of global mortality and disability worldwide. Machine learning (ML) models enhance prognostic accuracy by analysing complex, multidimensional clinical data. The aim of this systematic review and meta-analysis is to identify the gaps in the current ML models, along with methodological and performance outcomes in AIS. Further, the study objective was to identify the most frequently used algorithms and compare their relative effectiveness, thereby supporting future research to develop novel ML-based predictive models for stroke care management.

**Methods:**

The systematic review followed PRISMA guidelines with PROSPERO registration. A comprehensive search was performed in PubMed, Scopus, and Web of Science using MeSH keywords. Data extraction captured study characteristics, ML algorithms, and outcome metrics. We used the PROBAST and TRIPOD-AI to assess the qualities and bias of included studies. Meta-analysis of AUC values across ML models were conducted to synthesize model performance used a random-effects model to summarize and analyse the data and assessed heterogeneity (*I*^2^) statistic using SPSS-29 and R-Studio-4.2.0.

**Results:**

A total of 14 studies were included in the systematic review, with 12 eligible for meta-analysis. The pooled AUC of ML models was 0.87 (95% CI, 0.83–0.91), demonstrating strong predictive performance despite substantial heterogeneity (*I*^2^ = 99%). Random forest (RF) (AUC = 0.85) and SVM (AUC = 0.82) outperformed logistic regression (LR) (AUC = 0.75), while XGBoost showed stable performance (AUC = 0.82); heterogeneity was mainly driven by study design, publication year, and algorithm type (*p* < 0.001).

**Conclusion:**

ML-based models show potential for improving prognostic assessment in AIS; however, substantial heterogeneity and methodological limitations across studies limit the generalizability of pooled performance estimates.

**Systematic review registration:**

https://www.crd.york.ac.uk/PROSPERO/view/CRD420251033217, (Registration number: CRD420251033217).

## 1 Introduction

Stroke is a neurological condition caused by an interruption of blood flow to the brain, leading to neuronal injury and functional impairment (1). According to the World Health Organization (WHO), stroke is the second leading cause of death and the third leading cause of disability worldwide, contributing to a major global health burden. Annually, 15 million people worldwide suffer a stroke, among them, nearly 5 million die, and another 5 million live with permanent disabilities, resulting in a significant burden on families, healthcare systems, and communities (2, 3). This global impact is evident in India, which accounts for nearly 10% of the worldwide stroke burden (4).

Ischemic stroke is the most common type of stroke, accounting for about 67.3 to 80.5% of all cases (5). It occurs due to a sudden reduction or blockage of cerebral blood flow, leading to brain tissue ischemia. This reduction in blood flow is most often caused by arterial thrombosis or embolism, commonly associated with atherosclerosis, cardiac disorders, or small vessel disease (6). The major risk factors influencing the development of these clinical conditions include modifiable risks such as high blood pressure, diabetes, high cholesterol, smoking, obesity, and physical inactivity and non-modifiable factors such as older age, male sex, and a family history of stroke (7, 8).

Early detection and assessment of ischemic stroke are essential for improving patient prognosis (9). National Institutes of Health Stroke Scale (NIHSS) tools are widely used to assess the severity of neurological impairment and to guide treatment decisions. However, these traditional methods have limitations in predicting long-term functional recovery (10, 11). To address these challenges, ML techniques are increasingly being utilized to analyse complex clinical data, providing more accurate prognostic predictions and supporting personalized treatment strategies in ischemic stroke management (12, 13).

In recent years, ML has been increasingly applied across various scientific fields, especially in medical diagnosis and prognosis prediction (14, 15). It is now widely used in the field of stroke research to analyse large and complex datasets, providing deeper insights into disease mechanisms and improving the prediction of clinical outcomes (16, 17). Several studies have used ML techniques to predict outcomes in patients with AIS. Although, existing evidence is limited and heterogeneous across datasets, algorithms, and validations, with small samples and inconsistent reporting reducing the robustness and clinical relevance of the findings.

Therefore, we aimed to conduct a systematic review and meta-analysis to identify gaps in the current ML models, along with methodological and performance outcomes in AIS. Further, the study objective was to identify the most frequently used algorithms and compare their relative effectiveness through AUC analysis, thereby supporting future research to develop novel ML-based predictive models for stroke care management.

## 2 Methodology

### 2.1 Protocol and study registration

The study was conducted in accordance with Preferred Reporting Items for Systematic Reviews and Meta-Analyses (PRISMA) guidelines (18). The protocol was registered with the International Prospective Register of Systematic Reviews (PROSPERO ID: CRD420251033217).

A comprehensive literature search was conducted across three major databases, PubMed, Scopus, and Web of Science to identify relevant studies published between 2010 and 2024. Only articles published in English were considered for study. To optimize the retrieval of relevant studies, a combination of Medical Subject Headings (MeSH) terms and free-text keywords was employed. These were combined using Boolean operators (AND, OR, NOT) to enhance the search strategy as shown in Table 1.

**Table 1:** Search strategy applied across selected databases using predefined keywords and MeSH terms.

Databases	Search strategy
PubMed/MEDLINE	[(“AIS” OR “Acute stroke”) AND (“ML” [MeSH] OR “Artificial intelligence” [MeSH] OR “ML” OR “Artificial intelligence”) AND (“Prediction” OR “Prognosis” OR “Outcome” OR “Functional outcome”)]
Scopus	TITLE-ABS-KEY [(“AIS” OR “Acute stroke”) AND (“ML” OR “Artificial intelligence”) AND (“Prediction” OR “Prognosis” OR “Outcome” OR “Functional outcome”)]
Web of science	TS = (“AIS” OR “Acute stroke”) AND TS = (“ML” OR “Artificial intelligence”) AND TS = (“Prediction” OR “Prognosis” OR “Outcome” OR “Functional outcome”)

### 2.2 Eligibility criteria

Studies were considered eligible if they (1) included individuals diagnosed with AIS from diverse healthcare facilities, (2) employed ML models such as LR, RF, support vector machine (SVM), or XGBoost to develop predictive models using clinical and demographic variables, (3) compared different algorithms and validation strategies, (4) evaluated using predictive performance metrices (AUC) or clinical assessments including the modified Rankin Scale (mRS) and NIH Stroke Scale (NIHSS), and (5) followed retrospective, cohort, cross-sectional, or other observational study designs published in English between 2010 and 2024. Studies were excluded if they (1) did not implement ML or compare model performance, (2) were not specific to AIS, (3) failed to report key outcomes, (4) lacked internal or external validation, (5) Published in languages other than English, (6) lacked full-text availability, and (7) overlapping or duplicate datasets.

### 2.3 PICO search guide

#### 2.3.1 Population (P)

Individuals diagnosed with AIS.

#### 2.3.2 Intervention (I)

Application of ML techniques to predict clinical outcomes in AIS, utilizing algorithms such as LR, RF, SVM, XGBoost, neural networks, deep learning, and ensemble methods based on structured clinical and laboratory data.

#### 2.3.3 Comparator (C)

Traditional outcome prediction approaches or direct comparisons between different ML models.

#### 2.3.4 Outcome (O)

Primary outcomes focus on model predictive performance (AUC), while clinical outcomes are measured using functional and neurological assessments, including the mRS and the NIHSS.

### 2.4 Literature screening

All retrieved articles from the selected databases were initially assessed to identify and remove duplicate records, ensuring that only unique studies were retained for further evaluation. The remaining articles underwent a structured, multi-stage screening process based on predefined inclusion and exclusion criteria. Two independent reviewers (UK and VG) screened the unique records by examining titles and abstracts to assess their relevance. Any discrepancies in study eligibility were discussed and resolved by consensus, and if disagreements persisted, a third reviewer (NC) was consulted for a final decision. The initial screening of titles, abstracts, and potentially relevant full texts was conducted by one reviewer, while a second reviewer independently reassessed the shortlisted citations to ensure the reliability and consistency of the selection process. Studies identified as potentially eligible through the screening process were subsequently subjected to full-text assessment. Following full-text evaluation, a total of 14 studies met the predefined eligibility criteria. Of these, 12 studies were included in the quantitative meta-analysis, as they provided sufficient information for pooling of AUC estimates. The remaining two studies were excluded from the meta-analysis due to insufficient quantitative data, but were retained for qualitative synthesis to ensure a comprehensive assessment of the available evidence.

### 2.5 Data extraction

A standardized data extraction form was developed to systematically gather key characteristics and outcome-related information from each included study. Extracted data included the first author’s name, year of publication, country of origin, study design, source and nature of patient data (e.g., hospital records, national registries), and method of data collection. The type of stroke investigated was also recorded, with a specific focus on AIS. Further details encompassed population size, sample size, partitioning of datasets into training and testing sets, model validation approaches (internal and/or external), type of ML or statistical models employed, and predictive performance of the models. Information on clinical outcome assessment was also extracted, particularly the use of the mRS and NIHSS for evaluating functional outcomes post-stroke and calibration method.

Data extraction was independently conducted by two reviewers (SP and AS). Any discrepancies were resolved through discussion and consensus, with arbitration by a third reviewer (NC) when necessary.

### 2.6 Risk of bias and quality assessment

The methodological quality and potential risk of bias of the included studies were systematically assessed using the Prediction Model Risk of Bias Assessment Tool (PROBAST), it is specifically designed for the evaluation of prediction modeling studies. PROBAST assesses risk of bias and applicability across four key domains: participants, predictors, outcome, and analysis. Each domain was critically examined to identify methodological limitations that could influence the validity and reliability of reported predictive performance estimates. In addition, the applicability of the findings to the review question were assessed. This structured approach ensured a thorough and transparent evaluation of the internal validity of all included studies as presented in Supplementary Table 1.

For each PROBAST domain, judgements were assigned using standardized symbols: “+” for low risk, “−” for high risk, and “?” for unclear risk of bias due to insufficient or incomplete reporting. The use of these symbols facilitated consistent and transparent classification of methodological quality across studies.

The use of PROBAST enabled a robust and transparent appraisal of study quality, highlighting potential sources of bias that could impact the reliability of predictive performance outcomes. This was particularly relevant given that the majority of included studies employed retrospective designs and utilized ML algorithms, and frequently lacked external validation, all of which may increase the risk of bias in prediction modeling research.

For assessing the reporting quality of ML based prediction model studies, Collins and Moons (19) introduced ML specific extension of the Transparent Reporting of a Multivariable Prediction Model for individual Prognosis or Diagnosis (TRIPOD) statement, known as TRIPOD-AI. Based on this, reporting quality was evaluated using 12 key items adapted from the TRIPOD-AI framework.

Risk of bias evaluations were performed independently by two reviewers (VG and SP) to minimize bias. Ensuring methodological rigor and strengthening the overall reliability of the quality assessment process.

### 2.7 Outcome measures

The primary outcome was the area under the curve (AUC). It was selected as the main pooled metric because it provides a robust assessment of the discriminative performance of ML models in predicting outcomes in patients with AIS.

Secondary outcomes included additional performance metrics, including accuracy, sensitivity, and specificity, which provided additional assessment of model performance. Clinical outcomes included functional recovery, measured using the mRS, and neurological status, assessed with the NIHSS, reflecting the outcomes predicted by the models.

### 2.8 Data synthesis and statistical analysis

The extracted data were synthesized using both qualitative and quantitative approaches to provide a comprehensive evaluation of the included studies. Study characteristics were systematically summarized, and model performance metrics were pooled where appropriate. The AUC was the primary measure of discriminative performance for the ML models. When 95% confidence intervals (CI) or standard errors (SE) for AUC were not reported, they were estimated using the normal approximation method.

Given that AUC values are bounded between and 0 and 1, AUC estimates were logit- transformed prior to pooling to stabilize variances and better satisfy the assumptions of meta- analytic models. Following meta-analysis, pooled estimates and corresponding CI were back-transformed to the original AUC scale to facilitate interpretation.

Considering the variability in predictor variables, feature selection techniques, and modeling algorithms across studies, a random-effects meta-analysis model was used to pool AUC estimates, accounting for between-study heterogeneity.

Heterogeneity across studies was evaluated using the *I*^2^ statistic, with thresholds of 25, 50, and 75% indicating low, moderate, and high heterogeneity, respectively, as recommended in the Cochrane Handbook (20). To assess potential publication bias, both qualitative and quantitative approaches were employed: funnel plots were visually examined for asymmetry, and Egger’s regression test was conducted, with *p* < 0.05 indicating significant bias. To further investigate the sources of heterogeneity, subgroup analyses were performed based on study location, publication year, study design, and algorithm type. All statistical analyses were carried out using IBM SPSS Statistics (version 29) and R studio (version 4.2.0) to ensure robustness and reproducibility.

## 3 Results

### 3.1 Literature search

**Figure 1:**
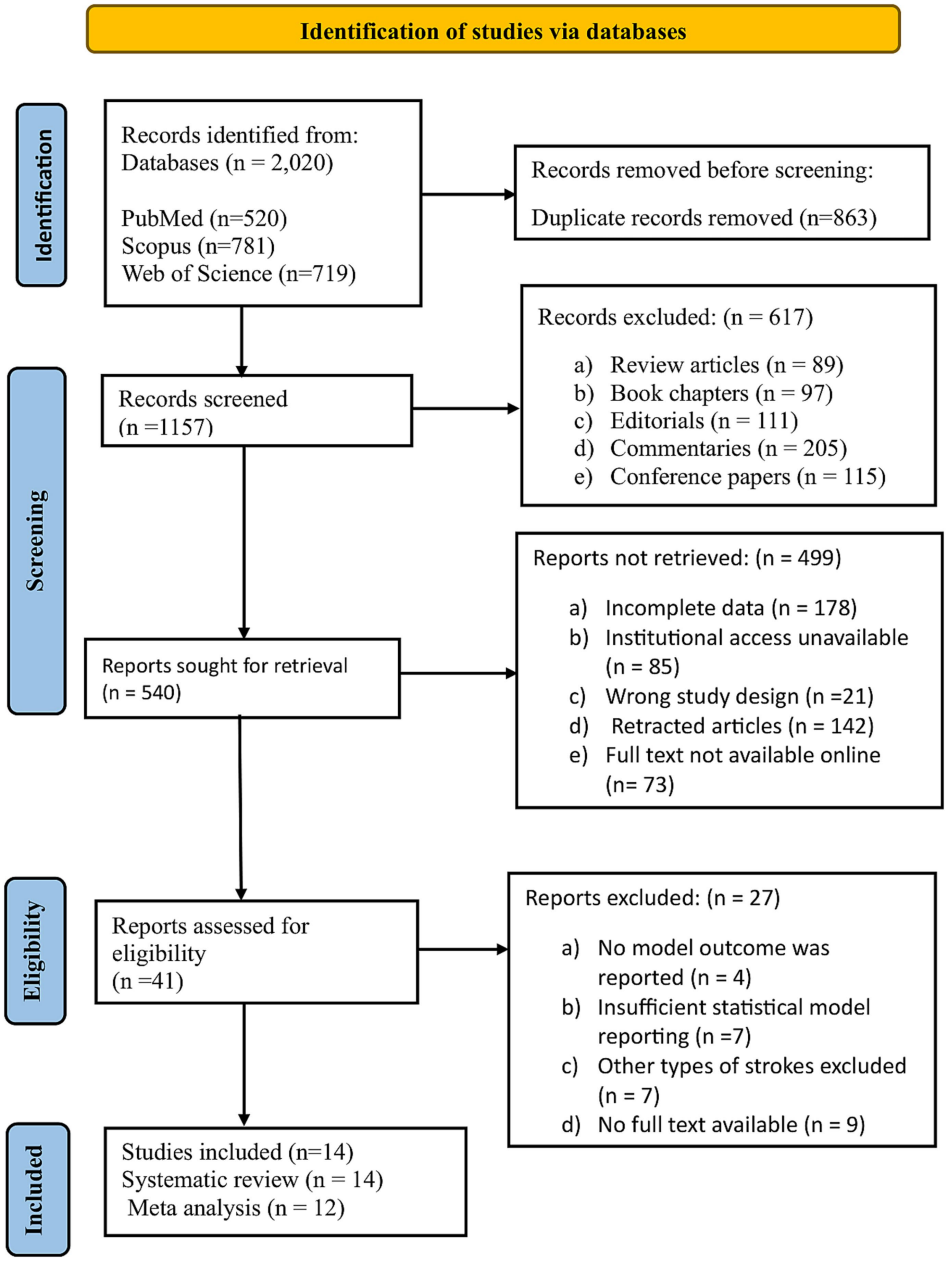
PRISMA flowchart of study selection and screening.

A total of 2,020 records were identified from PubMed (520), Scopus (781), and Web of Science (719). After removing 863 duplicates, 1,157 unique records were screened, and 617 were excluded as review articles, book chapters, editorials, commentaries, and conference proceedings. From the 540 reports selected for retrieval, 499 were not accessible due to incomplete data, institutional access unavailable, wrong study design, retracted articles, and full text not available online. The remaining 41 reports were assessed for eligibility, with 27 excluded because they did not report outcomes, had insufficient statistical reporting, included other types of strokes, or had no full text available. Finally, a total of 14 studies were included in the systematic review, of which 12 were eligible for inclusion in the meta-analysis, as shown in Figure 1.

**Figure 2:**
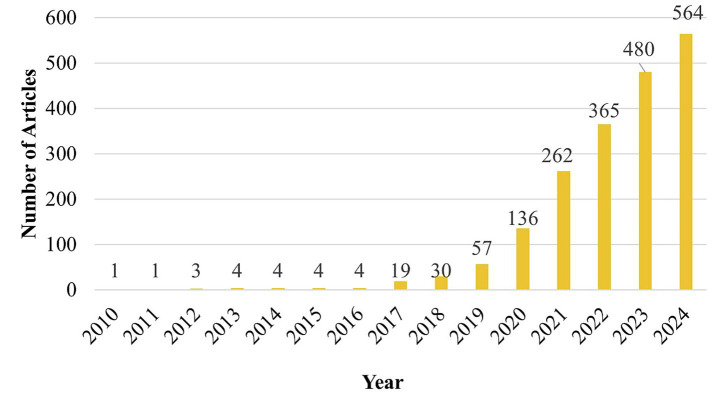
Annual growth of publications on machine learning from 2010–2024.

**Figure 3:**
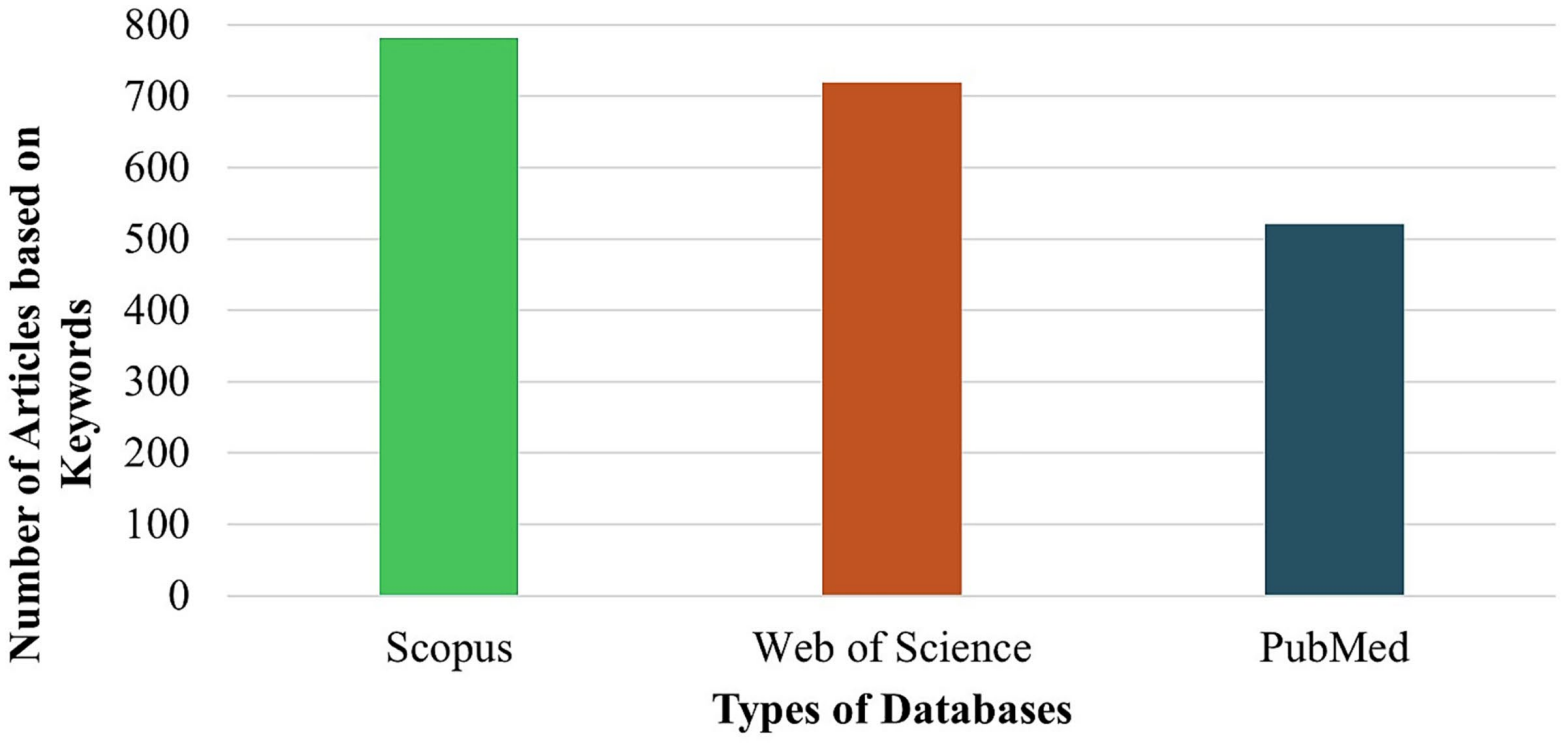
Comparative distribution of articles among key indexing databases.

**Figure 4:**
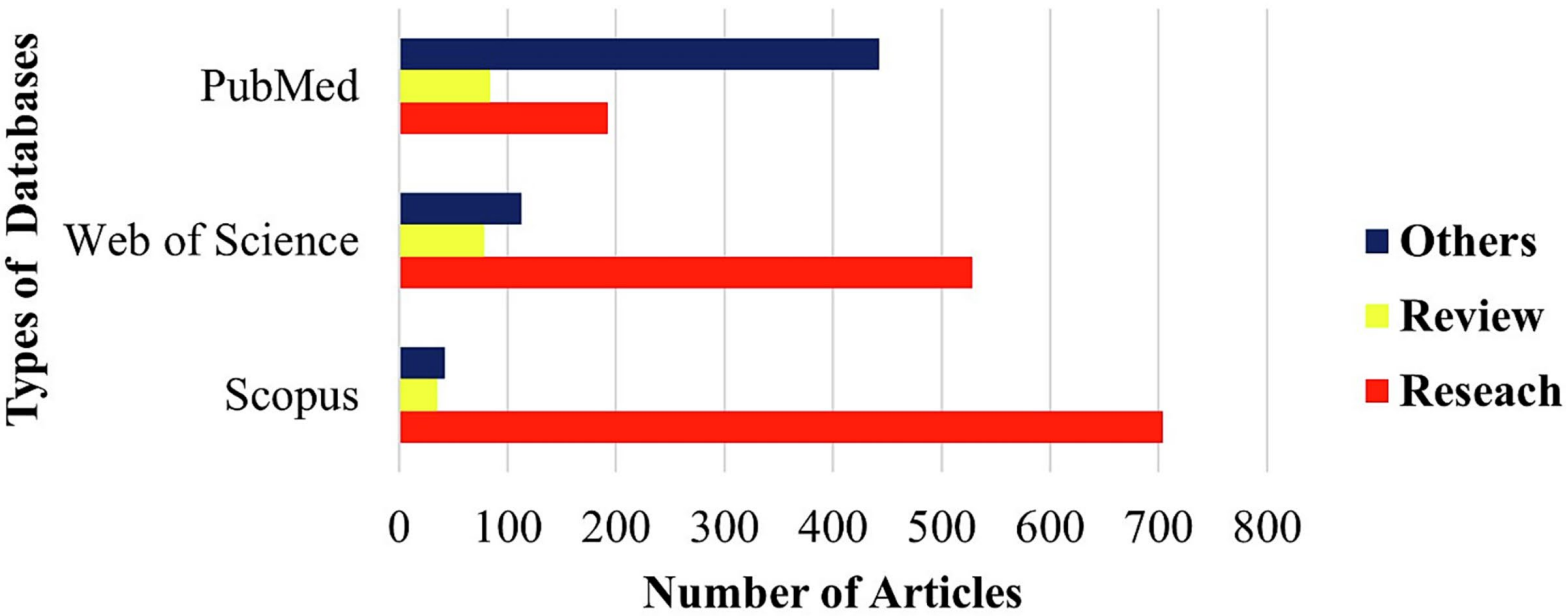
Distribution pattern of research, review, and other articles across academic databases.

Research publications in the field of stroke have experienced substantial growth over the past decade. Between 2010 and 2016, the annual number of published articles remained relatively low, ranging from just 1 to 4 articles per year. However, starting from 2020, there was a notable increase in research progress, with the number of publications increasing rapidly. By 2024, the annual publication count reached a peak of 564 articles, representing a significant rise from 136 articles in 2020. This observation highlights the reflecting of growing focus and progress in stroke research, as summarized in Figure 2. The analysis of database coverage based on keywords revealed that Scopus retrieved the highest number of articles, with approximately 780 publications. This was followed by Web of Science, contributing around 719 articles, while PubMed included a comparatively lower total of about 520 publications, as shown in Figure 3. The distribution of publication types indicates that original research articles constitute the majority across all databases. Scopus had the highest number of research papers, with approximately 704 articles, followed by Web of Science, which included around 528 research publications. In comparison, PubMed contained a relatively larger proportion of non-research (Other) articles, totalling about 443 publications. Review articles were comparatively less common, suggesting that most contributions are based on primary research data. This observation is further detailed in Figure 4, which highlights the focus of publications within these databases.

**Figure 5:**
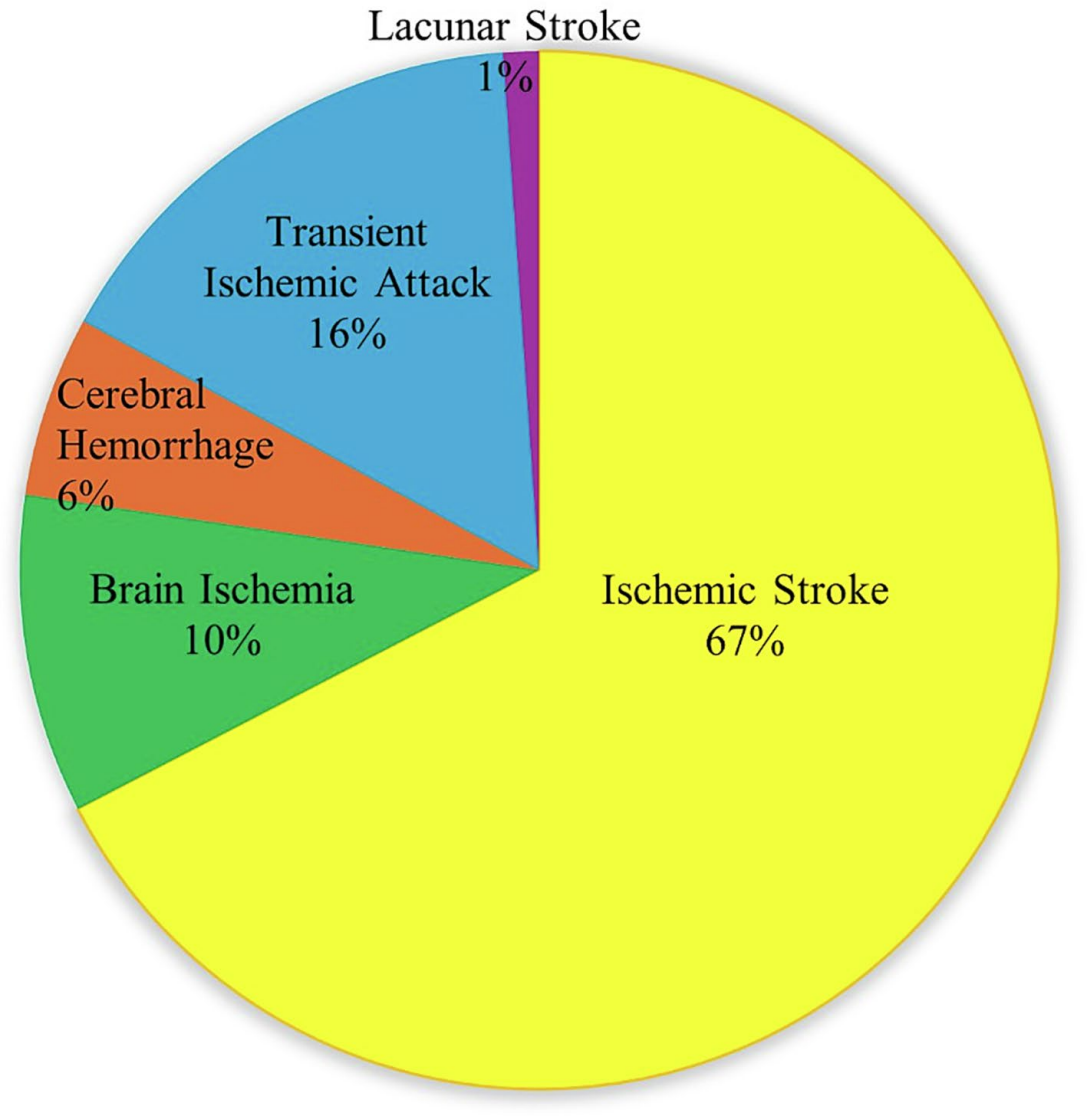
Publication-based distribution of stroke subtypes.

**Figure 6:**
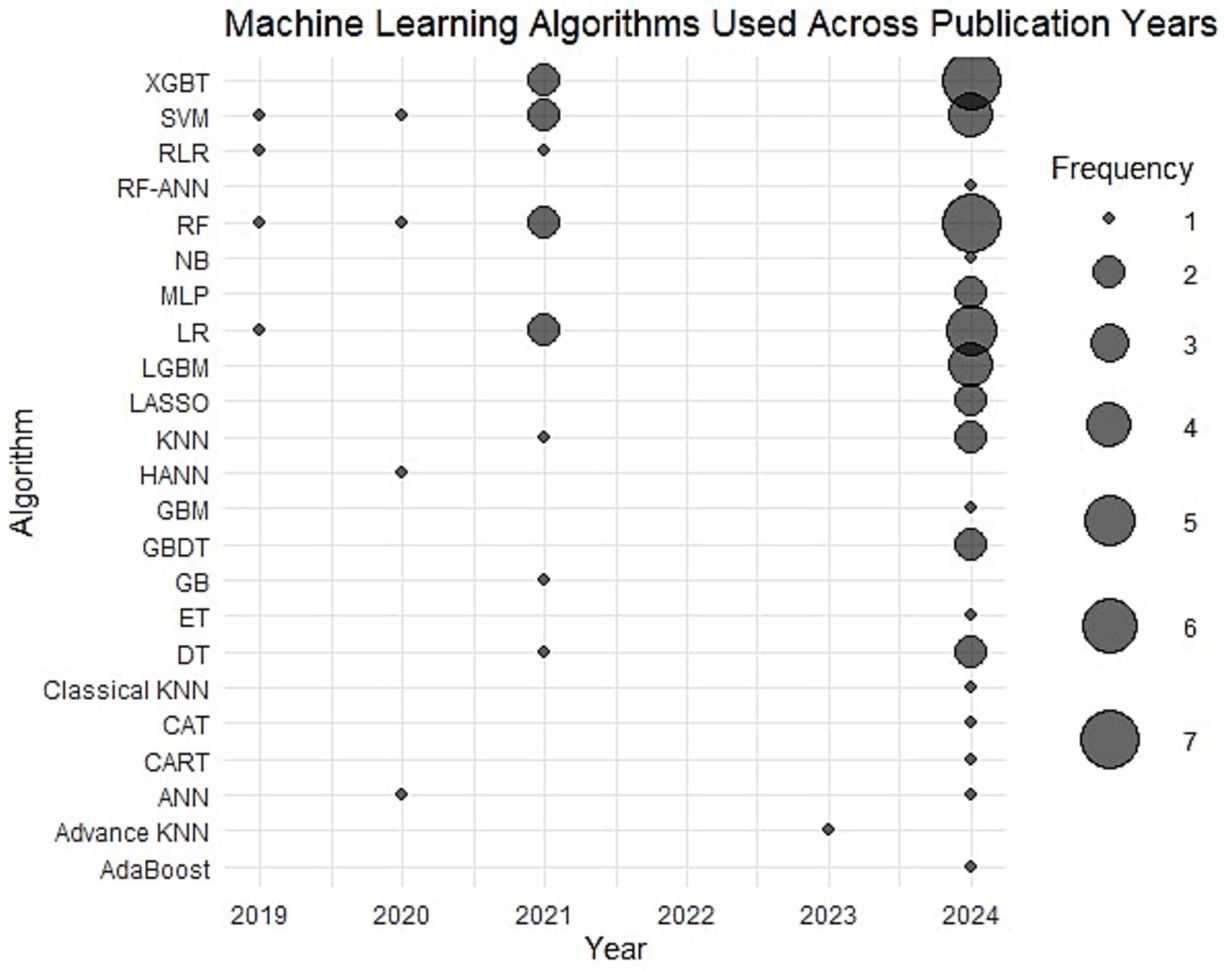
Frequency pattern in machine learning algorithm from 2019 – 2024.

In stroke research, different stroke subtypes have been investigated with varying emphasis. During the initial literature search across all databases, Ischemic stroke-related articles accounted for 67% of the retrieved articles. Brain ischemia accounts for 10%, while transient ischemic attack (TIA) is covered of 16%. Less studied are cerebral hemorrhage at 6% and lacunar stroke at 1%, as shown in Figure 5. From 2019 to 2024, the use of ML models in stroke research increased overall, with variation across years. In 2019, a limited set of algorithms was reported, including SVM, RLR, RF, and LR, each in one study. In 2020, SVM and RF were the most frequently applied models, each appearing in two studies. By 2021, model diversity increased, with XGBT, SVM, RF each used twice in study, RLR, AND KNN each used in one study, highlighting broader methodological adoption. A slight decline in model usage was observed in 2022. In 2023, the focus shifted toward more specialized approaches, with classical KNN reported once. The highest level of model usage occurred in 2024, where RF and XGBT was used seven times, followed by SVM four times. This shows that ML methods are being used more often and in more ways in stroke research, as depicted in Figure 6.

### 3.2 Risk of bias assessment

The high risk of bias observed using PROBAST across the included studies was primarily attributable to methodological limitations within the analysis domain, including small sample sizes, predominantly retrospective study designs, and the absence of independent validation cohorts. Such limitations reduce the robustness and generalizability of the developed prediction models. Furthermore, the lack of validation datasets increases the likelihood of model overfitting and optimistic performance estimates. However, applicability assessments revealed low concern across all domains, suggesting that the study populations, predictors, and outcomes were appropriate and relevant to the review objectives as presented in Table 2.

**Table 2:** Methodological quality assessment of included studies.

Study	Risk of bias				Applicability			Overall	
	Participants	Predictors	Outcome	Analysis	Participants	Predictors	Outcome	ROB	Applicability
Abujaber A. et al., 2024 (1)	+	+	?	−	+	+	+	−	+
Nishi H. et al., 2019 (4)	+	+	+	−	+	+	+	−	+
He Y. et al., 2024 (5)	?	+	+	−	+	+	+	−	+
Huang Q. et al., 2024 (7)	+	+	+	−	+	+	+	−	+
Brugnara G. et al., 2020 (8)	?	+	?	?	+	+	+	?	+
Xing Y. et al., 2024 (9)	+	+	+	−	+	+	+	−	+
Xu L. et al., 2024 (10)	+	+	?	−	+	+	+	−	+
Mbarek L. et al., 2024 (11)	+	+	+	−	+	+	+	−	+
Bamodu O. A. et al., 2024 (12)	?	+	+	?	+	+	+	?	+
Lin C. H. et al., 2020 (13)	?	+	+	−	+	+	+	−	+
Park D. et al., 2021 (14)	+	+	+	−	+	+	+	−	+
Shao H. et al., 2023 (15)	?	+	+	−	+	+	+	−	+
Wang X. et al., 2024 (16)	+	+	+	−	+	+	+	−	+
Abedi V. et al., 2021 (17)	+	+	?	−	+	+	+	−	+

(+) symbol low risk; (−): high risk; (?): unclear.

The adherence to TRIPOD-AI reporting items was high for study design, population characteristics, and model development, with most studies adequately reporting discrimination (AUC) and validation methods. However, reporting of calibration, handling of missing data, and model availability was limited as presented in Supplementary Table 2.

### 3.3 Study characteristics of included studies

A total of 14 studies were included in the meta-analysis, published between 2010 and 2024, and carried out across several countries: China (7 studies), Japan (1), Taiwan (2), South Korea (1), Qatar (1), Germany (1), and one joint study from the United States and Austria. Most studies had retrospective cohort or observational designs (1, 4, 7, 10–14, 16, 17), while others used retrospective study designs (8, 9), cross-sectional approaches (5), or were hospital-based pilot studies (15). With reference to data sources, nine studies were single-centre (1, 5, 9–13, 15, 16), and five were multicenter (4, 7, 8, 14, 17). The predominant stroke subtype studied was AIS (7–12, 14–17), while specific subtypes such as AIS with large vessel occlusion (LVO) were investigated in Nishi et al. (4), and broader classifications such as ischemic stroke (IS) were reported in Abujaber et al. (1) and He et al. (5); one study uniquely included both ischemic and hemorrhagic stroke (13). The mean age of participants varied across the included studies, ranging from 44.3 ± 5.5 years (11) to 74.8 ± 11.6 years (4), with intermediate values reported in several studies, including 54.2 ± 13.6 years (1), 64.01 ± 11.31 years (5), 65.8 ± 11.3 years (14), 67 ± 13 years (17), 68.34 ± 12.36 years (7), 68.5 ± 13.7 years (12), and 68.37 ± 13.38 years (10); while, some studies did not provide information on mean age (8, 9, 13, 15, 16).

In most studies, the follow-up period was set at 3 months (90 days) (1, 4, 7–16), two studies deviated from this timeline: one assessed outcome from admission until hospital discharge (5), while another extended the follow-up to 5 years (17), providing insights into both short- and long-term outcomes. NIHSS scores were reported with considerable variation across the studies. Huang et al. (7) reported an interquartile range (IQR) of 4.00 (3.00–7.00), while Abujaber et al. (1) indicated an IQR of 5, SD of 6. In Nishi et al. (4), the derivation cohort showed an IQR of 19 (14–23) and the validation cohort an IQR of 20 (14–24). Xing et al. (9) also presented an IQR of 4.00 (3.00–7.00). The median NIHSS score was 6.00 with an IQR of 4.00–11.75 in Wang et al. (16). Shao et al. (15) reported an IQR of 7 (1–20) for favorable outcomes and 13 (4–23) for unfavorable outcomes. In Park et al. (14), the mean NIHSS was 2.3 ± 3.2, whereas Bamodu et al. (12) recorded a mean of 7.5 ± 7.9. Mbarek et al. (11) reported a median score of 2 with an IQR of 1–4. Several studies did not provide NIHSS data (5, 8, 10, 13, 17). Functional outcomes were primarily assessed using the mRS. In most studies, favorable outcomes were defined as mRS ≤ 2 and unfavorable outcomes as mRS > 2 (5, 7, 12–16) and one study reported mRS < 2 as favorable and mRS > 2 (4). Some studies used different thresholds, with Xing et al. (9) applying mRS ≤ 3 as favorable and mRS > 3 as unfavorable, while Mbarek et al. (11) reported unfavorable outcomes as mRS ≥ 3. A few studies did not provide mRS-based outcomes (1, 8, 10, 17), which limited comparability across studies. In addition to this calibration performance was reported in only a subset of studies. Two studies assessed calibration using the Brier score (1, 7), while four studies evaluated calibration through calibration curves or plots (9, 11, 14, 16). The remaining studies did not report any calibration assessment (4, 5, 8, 10, 12, 13, 15, 17) as presented in Table 3.

**Table 3:** General study characteristics of included studies.

First author, publication year	Country	Study design	Data source	Stroke type	Age (mean ± SD)	Follow up duration	NIHSS scores IQR/SD	mRS (modified Ranking Scale) outcome		Calibration method
								Favorable outcome	Unfavorable outcome	
Abujaber A. et al., 2024 (1)	Qatar	Retrospective cohort study	Single centre	IS	54.2 ± 13.6	3 months (90 days)	IQR = 5 SD = 6	NA	NA	Brier score
Nishi H. et al., 2019 (4)	Japan	Retrospective observational cohort study	Multicentre	AIS with LVO	74.8 ± 11.6	3 months (90 days)	IQR = 19 (14–23) in the derivation cohort, and IQR = 20 (14–24) in the validation cohort	mRS < 2	mRS > 2	NA
He Y. et al., 2024 (5)	China	Cross-sectional study	Single centre	IS	64.01 ± 11.31	From admission to discharge	NA	mRS ≤ 2	mRS > 2	NA
Huang Q. et al., 2024 (7)	China	Retrospective observational study	Multicentre	AIS	68.34 ± 12.36	3 months (90 days)	IQR = 4.00 (3.00–7.00)	mRS ≤ 2	mRS > 2	Brier score
Brugnara G. et al., 2020 (8)	Germany	Retrospective study	Multicentre	AIS	NA	3 months (90 days)	NA	NA	NA	NA
Xing Y. et al., 2024 (9)	China	Retrospective study	Single centre	AIS	NA	3 months (90 days)	IQR = 4.00 (3.00–7.00)	mRS ≤ 3	mRS > 3	Calibration curve plot
Xu L. et al., 2024 (10)	China	Retrospective cohort study	Single centre	AIS	68.37 ± 13.38	3 months (90 days)	NA	NA	NA	NA
Mbarek L. et al., 2024 (11)	China	Retrospective cohort study	Single centre	AIS	44.3 ± 5.5	3 months (90 days)	Median (IQR) = 2 (1–4)	mRS ≤ 2	mRS ≥ 3	Calibration curve plot
Bamodu O. A. et al., 2024 (12)	Taiwan	Retrospective cohort study	Single centre	AIS	68.5 ± 13.7	3 months (90 days)	Mean ± SD = 7.5 ± 7.9	mRS ≤ 2	mRS > 2	NA
Lin C. H. et al., 2020 (13)	Taiwan	Retrospective cohort study	Single centre	Ischemic and Hemorrhagic stroke	NA	3 months (90 days)	NA	mRS ≤ 2	mRS > 2	NA
Park D. et al., 2021 (14)	South Korea	Retrospective cohort study	Multicentre	AIS	65.8 ± 11.3	3 months (90 days)	Mean ± SD = 2.3 ± 3.2	mRS ≤ 2	mRS > 2	Calibration plot
Shao H. et al., 2023 (15)	China	Hospital-based pilot study	Single centre	AIS	NA	3 months (90 days)	IQR = 7 (1–20) for favorable outcomes; IQR = 13 (4–23) for unfavorable outcomes.	mRS ≤ 2	mRS > 2	NA
Wang X. et al., 2024 (16)	China	Retrospective observational study	Single centre	AIS	NA	3 months (90 days)	Median (IQR): 6.00 [4.00, 11.75]	mRS ≤ 2	mRS > 2	Calibration curve plot
Abedi V. et al., 2021 (17)	United States and Austria	Retrospective observational study	Multicentre	AIS	67 ± 13	5 years	NA	NA	NA	NA

AIS, acute ischemic stroke; IS, ischemic stroke; LVO, large vessel occlusions; NA, not available; SD, standard deviation; IQR, inter quartile range; mRS, modified Ranking Scale.

Table 4 presents an overview of the characteristics of the included studies, with sample sizes varying widely from smaller cohorts of 189 (15) and 218 (16) participants to larger datasets of 8,183 (1) and 35,798 (13) individuals. Generally, studies with larger sample sizes showed more stable and reliable model performance, whereas smaller datasets exhibited greater variability and a higher risk of overfitting.

**Table 4:** Study characteristics and performance measure of ML algorithms.

First author, publication year	Population size	Sample size	Predictors/variables/features	Testing data (%)	Training data (%)	Validation type	Algorithms used	AUC	Best model	AUC
Abujaber A. et al., 2024 (1)	15,859	8,183	Patient demographics, medical history, vital signs, laboratory results, NIHSS score, dysphagia screening, comorbidities, and treatment information	20	80	Internal validation	ANN	0.94	ANN	0.94
							SVM	0.86		
							XGBT	0.86		
							RF	0.87		
							LR	0.81		
Nishi H. et al., 2019 (4)	502	502	Demographic: age, sex, premorbid mRS, comorbidities, antithrombotic use. Clinical: stroke onset pattern, NIHSS, blood glucose, occlusion site/side, ASPECTS, IV-tPA, time metrics (last known well, hospital arrival)	10	90	10-fold cross-validation (internal validation)	LR	0.56	RLR	0.9
							RLR	0.90		
							SVM	0.89		
							RF	0.87		
He Y. et al., 2024 (5)	964	964	Blood biomarkers, clinical measures (NIHSS, mRS, blood pressure, risk level), and history of cerebral infarction	30	70	Five-fold and 10-fold cross-validation	CART	NA	RF	0.9
							ET	0.90		
							KNN	NA		
							LightGBM	0.89		
							LR	NA		
							RF	0.90		
							SVM	NA		
							CAT	0.91		
							XGBoost	NA		
Huang Q. et al., 2024 (7)	659	659	WBC, HCY, D-dimer, baseline NIHSS, FDP, and GLU	70	30	10-fold cross-validation	XGBoost	0.84	RF	0.85
							LR	0.85		
							LightGBM	0.66		
							RF	0.87		
							AdaBoost	0.83		
							DT	0.71		
							GBDT	0.74		
							MLP	0.63		
							SVM	0.77		
Brugnara G. et al., 2020 (8)	317	246	Baseline demographics, clinical characteristics, imaging parameters (ASPECTS, collateral status, clot burden), and treatment-related variables	20	80	Cross-validation	NA	NA	NA	NA
Xing Y. et al., 2024 (9)	1,856	Post-clustering cohorts: 139 and 122	Routine laboratory test within 2 h of admission	30	70	Post-clustering cross-validation and External validation	LASSO	0.82	RF	0.81
							DT	NA		
							XGBoost	NA		
							RF	0.81		
							RF-ANN	NA		
Xu L. et al., 2024 (10)	1,876	1,633	Blood counts, coagulation markers, blood chemistry analyses, and urine tests were conducted within 1 week of admission	15	85	10-fold cross-validation	LGBM	0.96	LGBM	0.96
							GBM	NA		
							RF	NA		
							KNN	NA		
							MLP	NA		
							NB	NA		
Mbarek L. et al., 2024 (11)	15,166	2,268	Demographic characteristics, thrombolytic therapy (alteplase), history of smoking, history of alcohol consumption, medical history, laboratory data, NIHSS	20	80	10-fold cross-validation	XGBT	0.80	XGBT	0.8
							GBDT	0.79		
							RF	0.79		
							LGBM	0.79		
							LR	0.78		
Bamodu O. A. et al., 2024 (12)	2,242	2,229	Demographic data - including age, sex, and the presence of cerebrovascular risk factors were collected on admission	NA	NA	10-fold cross-validation	XGBT	NA	XGBoost XGBT	NA
Lin C. H. et al., 2020 (13)	40,293	35,798	Demographic, clinical, laboratory	30	70	10-time repeated hold-out with 10-fold cross-validation	SVM	0.97	SVM	0.97
							RF	0.96		
							ANN	0.95		
							HANN	0.96		
Park D. et al., 2021 (14)	1,202	1,066	Demographic factors, stroke-related factors, laboratory findings, and comorbidities	30	70	10-fold cross-validation	RLR	0.86	RLR	0.86
							SVM	0.85		
							RF	0.82		
							KNN	0.82		
							XGBT	0.81		
Shao H. et al., 2023 (15)	202	189	Sex, cardioembolic risk factors, hypertension, and baseline NIHSS	30	70	Internal validation	Advanced KNN	0.88	Advanced KNN	0.88
							Classical KNN	0.75		
Wang X. et al., 2024 (16)	232	218	FIB, blood pressure-related markers, and TC, imaging technology	30	70	10-fold cross-validation	LR	0.77	LASSO	0.78
							XGBT	0.78		
							SVM	0.76		
							LASSO	0.78		
							RF	0.78		
Abedi V. et al.,2021 (17)	2,091	2,091	Clinical and retinal characteristics	20	80	10-fold repeated cross-validation	GB	0.71	RF	0.79
							RF	0.79		
							XGBoost	0.74		
							DT	0.73		
							SVM	0.63		
							LR	0.72		

WBC, white blood cell; HCY, homocysteine; NIHSS, National Institutes of Health Stroke Scale; FDP, fibrin degradation products; GLU, glucose; mRS, modified Rankin Scale; ASPECTS, Alberta Stroke Program Early CT Score; IV-tPA, intravenous tissue plasminogen activator; FIB, fibrinogen; TC, total cholesterol; RF, random forest; LR, logistic regression; XGBT, extreme gradient boosting tree; CART, classification and regression trees; SVM, support vector machine; RLR, regularized logistic regression; LASSO, least absolute shrinkage and selection operator; GB, gradient boosting; DT, decision tree; RF-ANN, random forest-artificial neural network hybrid; Advanced KNN, advanced k-nearest neighbors; Classical KNN, classical k-nearest neighbors; ANN, artificial neural network; HANN, hybrid artificial neural network; GBDT, gradient boosting decision tree; MLP, multilayer perceptron; LGBM, light gradient boosting machine; NA, not available.

The predictors used across various studies demonstrate considerable variability, common demographic and clinical variables include age, sex, comorbidities, and prior medical history, which are frequently incorporated into predictive models (1, 4, 8, 11–14). Laboratory biomarkers such as white blood cell count, D-dimer levels, fibrinogen, and coagulation indices are also routinely used, reflecting their significance in stroke prognosis (7, 9, 10, 16). Neurological assessment scores, including the NIHSS and the mRS, are integrated into several models to evaluate neurological deficits and functional outcomes (1, 4, 5, 11, 15). Imaging characteristics, such as the Alberta Stroke Program Early CT Score (ASPECTS), clot burden, and collateral circulation, are assessed in some studies to determine the extent of ischemic damage and vascular status (4, 8). Additionally, recent research has introduced emerging predictors like retinal parameters, emphasizing the multidimensional approaches employed for stroke outcome prediction (17). Overall, the diversity of predictors highlights the complexity of stroke prognosis and reflects ongoing efforts to refine predictive models for improved clinical decision-making.

Data partitioning and validation strategies vary across studies, the most common approach is a 70:30 split for training and testing, used in several studies (5, 7, 9, 13–16). Larger datasets often employ an 80:20 division (1, 8, 11, 17) while one study used an 85:15 split (10) and 10:90 split (4). A few reports did not specify the proportions (12). The most used validation technique is 10-fold cross-validation (4, 7, 10–12, 14, 16). Some studies utilized internal validation (1, 15), one study used a 10-time repeated hold-out with 10-fold cross-validation for very large cohorts (13), 10-fold repeated cross validation (17) and cross validation used in one study (8).

When assessing different ML methods, LR and its regularized variations were commonly employed (1, 4, 7, 11, 14). Among the most popular ensemble methods were gradient boosting techniques (AdaBoost, GBM, CAT, GB, XGBT, GBDT, LGBM, and GBM) (1, 5, 7, 9–12, 17) and RF (1, 7, 9, 11, 13). In addition to ensemble methods, tree-based models, such as (DT, CART, and ET) were evaluated in several studies (5, 7, 9, 17). In several studies, SVM model were used in Abujaber et al. (1), Nishi et al. (4), He et al. (5), Huang et al. (7), Lin et al. (13), Park et al. (14), Wang et al. (16), and Abedi et al. (17). In a number of investigations, neural network models such as ANN, HANN, RF-ANN models were applied (1, 9, 13). Specialized models like advanced and classical KNN (15), multilayer perceptrons (MLP), and LASSO regression (7, 9, 10, 16) were assessed less frequently.

Several included studies showed incomplete reporting of model performance metrics. Specifically, Brugnara et al. (8) did not report AUC values for any algorithm, while He et al. (5) and Xing et al. (9) lacked AUCs for several comparative models. In addition, Xu et al. (10) reported AUC only for LGBM, and Bamodu et al. (12) did not provide AUC for XGBoost.

Across the included literature, RF was most frequently identified as the highest- performing model (5, 7, 9, 17), while other studies found RLR (4, 14), LASSO (16), and advanced KNN (15) to be the best. Strong performance was also obtained by gradient boosting methods such as XGBT (11) and LGBM (10). One study favoured SVM (13), while ANN was optimal in one study (1). Overall, ensemble-based and neural network approaches demonstrated better predictive capacity for stroke outcomes.

### 3.4 Meta analysis and publication bias of model performance

**Figure 7:**
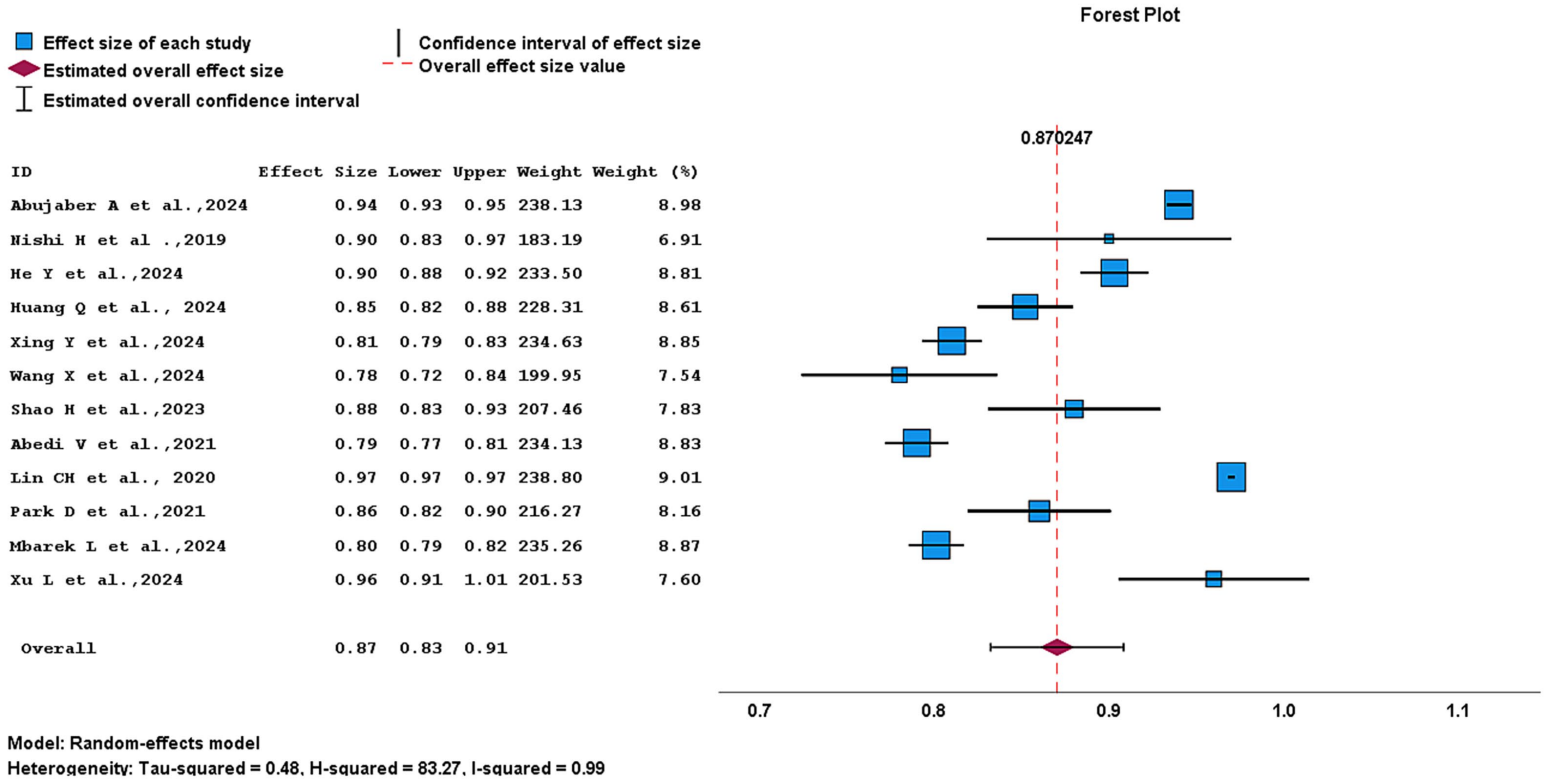
Forest plot of included studies assessing the best-performing ML algorithms.

**Figure 8:**
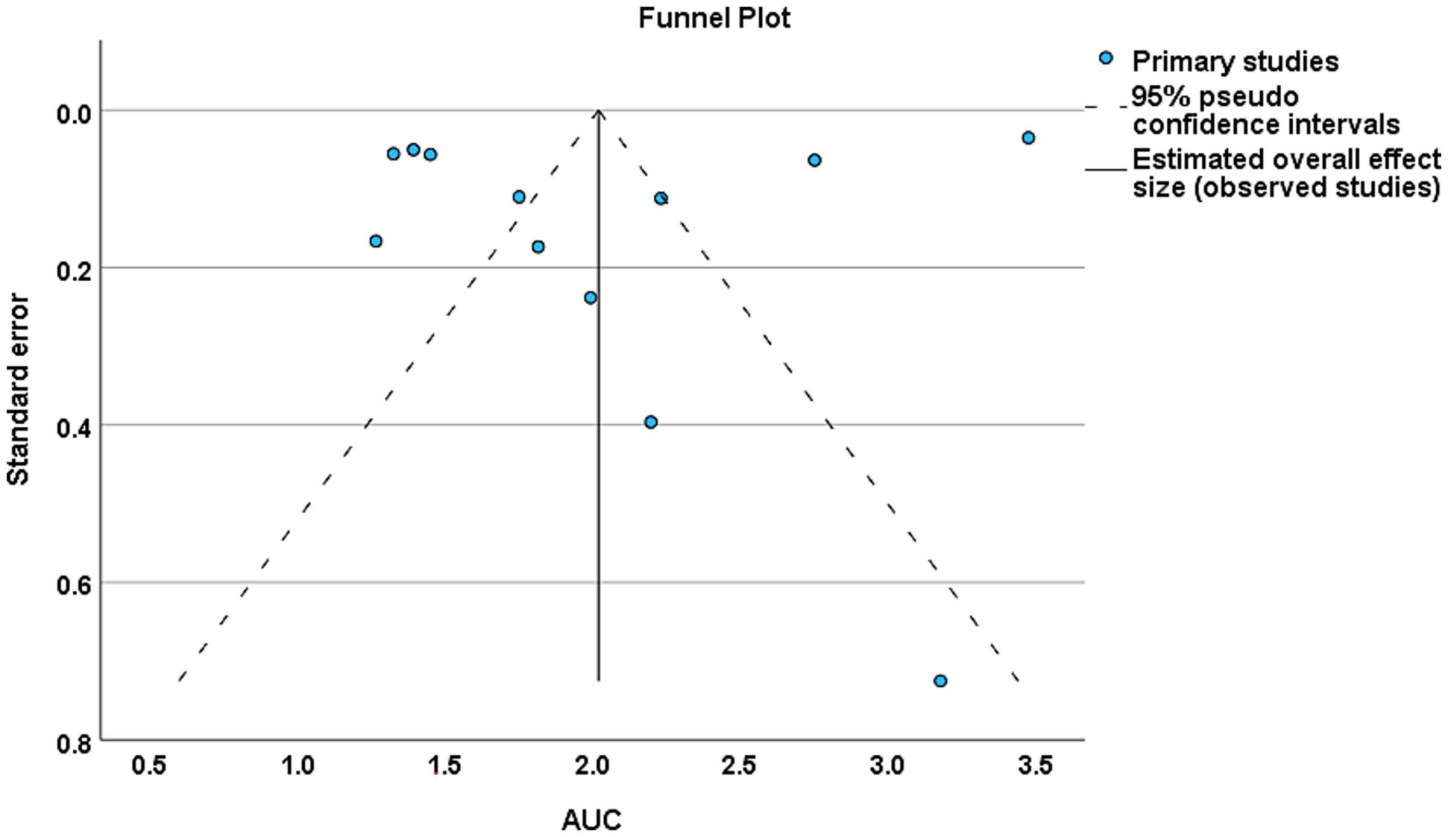
Funnel plot of studies on best-performing machine learning models for AIS, showing AUC with 95% CIs.

A total of 14 studies met the eligibility criteria and were included in the systematic review. Of these, 12 studies were eligible for quantitative synthesis and were therefore included in the meta-analysis (1, 4, 5, 7, 9–11, 13–17). The AUC was employed to assess model performance, with the top-performing models summarized in Figure 7. The pooled AUC was 0.87 (95% CI, 0.83–0.91), indicating strong overall predictive performance. A random-effect model was used to account for potential variability across studies. However, heterogeneity remained considerably high (*H*^2^ = 76.06; *I*^2^ = 99%), suggesting substantial differences between studies rather than random variation. In the forest plot, studies such as Nishi et al. (4) and Wang et al. (16) exhibited wider CIs, reflecting lower precision, while studies like He et al. (5) and Xing et al. (9) demonstrated narrower intervals, indicating higher precision. Some studies, including Abujaber et al. (1) and Lin et al. (13) showed extremely narrow intervals, appearing almost without visible CI with. Eight studies (1, 4, 5, 7, 10, 13–15) reported relatively high AUC (>0.85), contributing significantly to the pooled estimate, whereas four studies (9, 11, 16, 17) had lower AUC (<0.81), exerting less influence. Publication bias was examined using a funnel plot, as show in Figure 8, which revealed slight asymmetry and an uneven distribution of studies. Egger’s regression test supported this finding, showing a significant intercept (1.853, *p* < 0.001), indicating a potential risk of publication bias.

**Figure 9:**
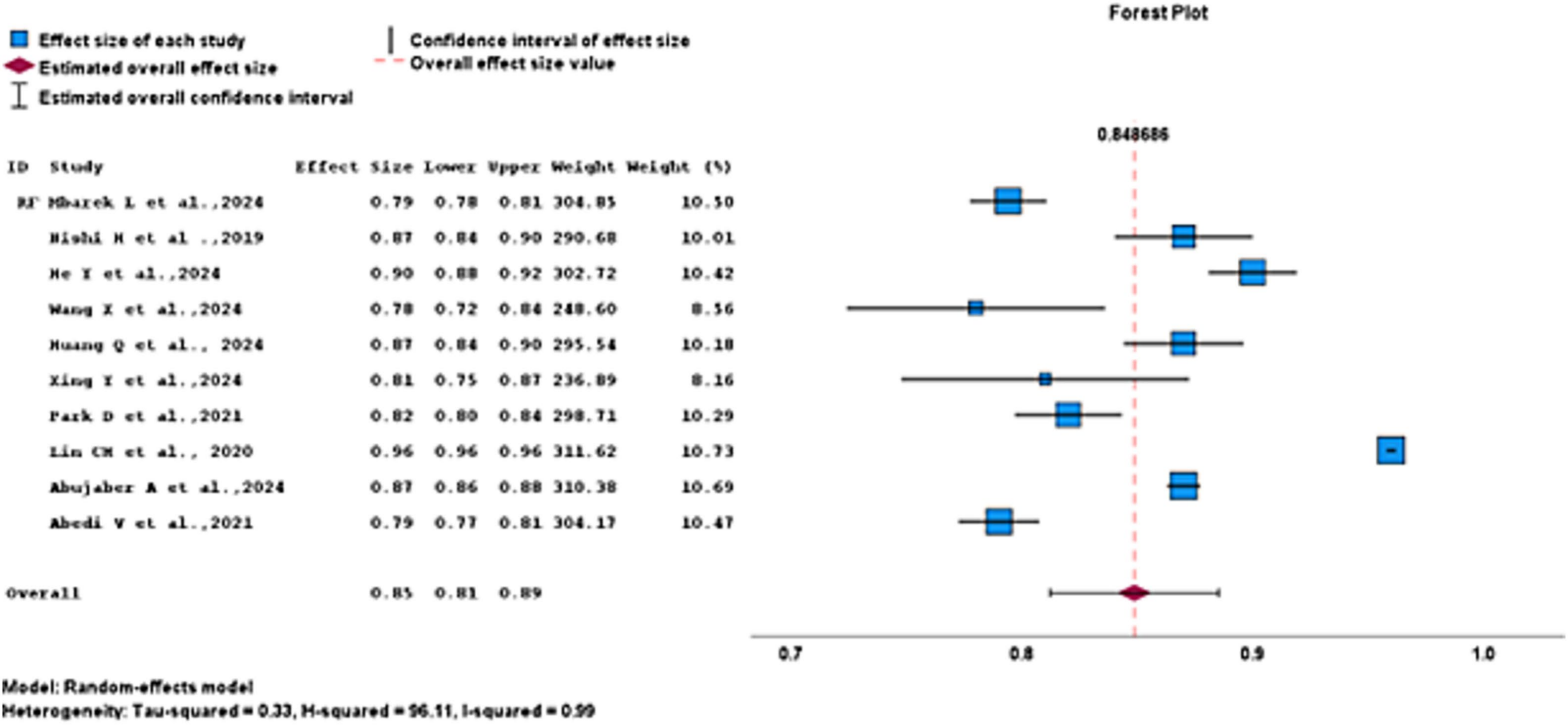
Random forest model for best performing ML algorithms.

The RF algorithm exhibited strong predictive performance for AIS, with a pooled AUC of 0.85 (95% CI, 0.81–0.89). Although a significant heterogeneity was observed among the studies (*H*^2^ = 96.11; *I*^2^ = 99%), reflecting considerable variability. The width of the CIs reflected the reliability of individual studies, with smaller studies such as Wang et al. (16) and Collins and Moons (19) showing broader intervals and larger, more precise studies like Abujaber et al. (1), He et al. (5), and Lin et al. (13) presenting narrower CIs. Overall, these results indicate that RF models provide consistent and reliable predictions of AIS outcomes across studies, as illustrated in Figure 9.

**Figure 10:**
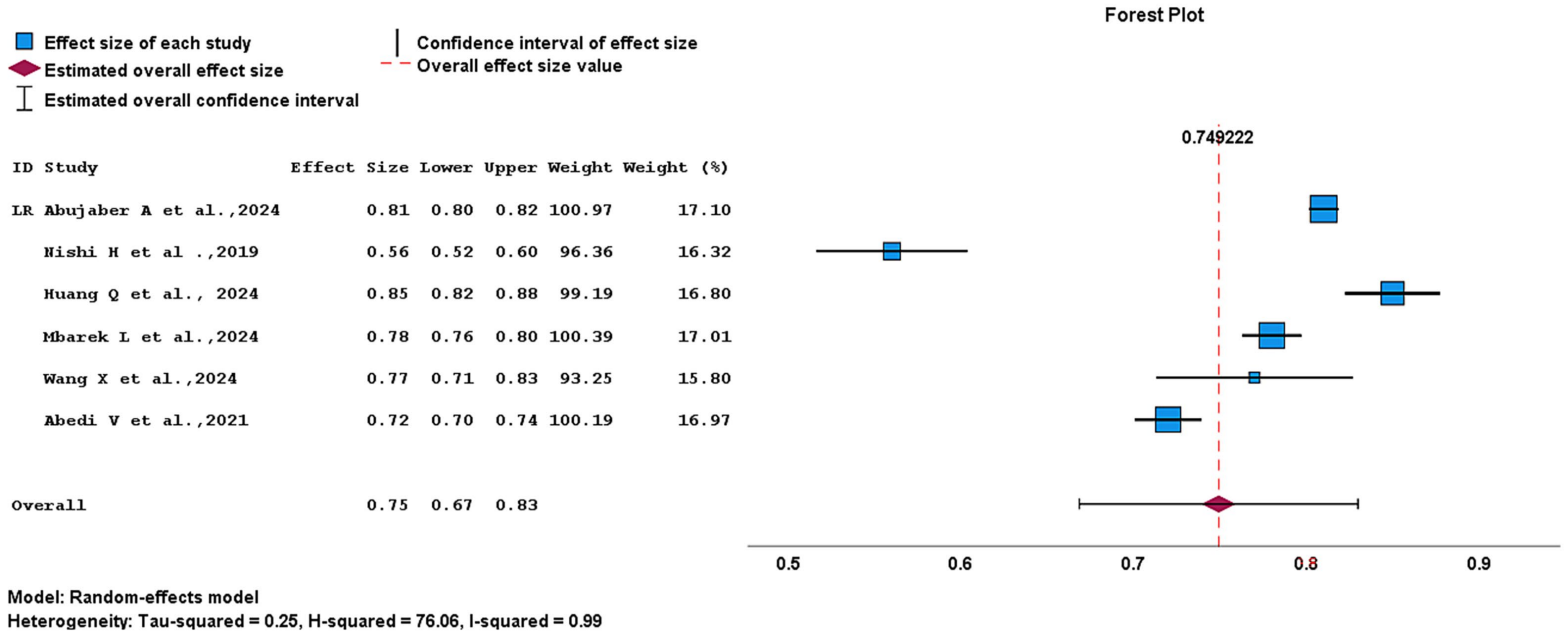
Forest plot of logistic regression models for outcome prediction in acute ischemic stroke.

**Figure 11:**
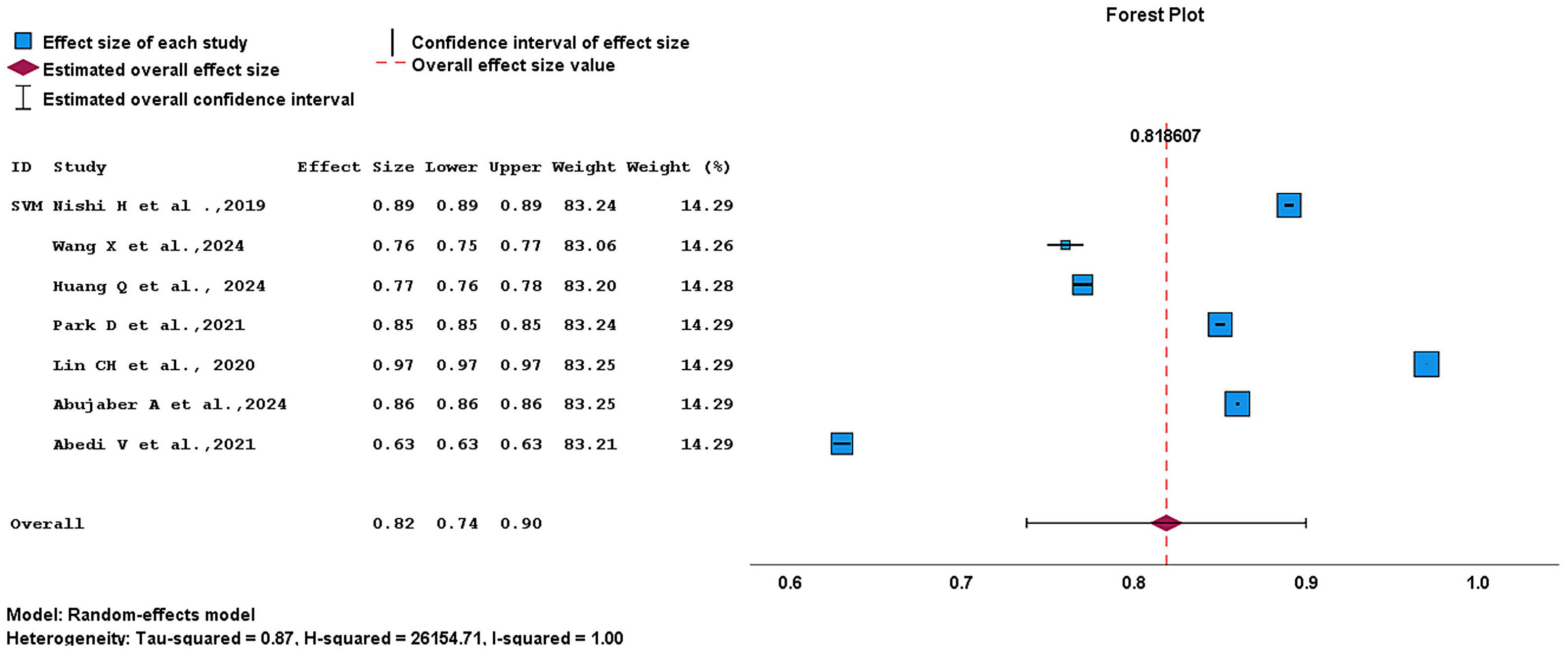
Forest plot illustrating outcome prediction using a SVM model in AIS.

A meta-analysis of LR model showed a pooled AUC of 0.75 (95% CI, 0.67–0.83). Among the included studies, Nishi et al. (4) demonstrated the widest CI (0.502–0.60), indication lower precision and smaller study weight. Whereas, studies such as Abujaber et al. (1) and Mbarek et al. (11) exhibited narrower CIs, reflecting more stable and precise effect estimate. Despite the application of a random-effect model, heterogeneity remained high (*I*^2^ = 99%; *H*^2^ = 76.06), indicating substantial variability across studies as presented in Figure 10. As for SVM models, the pooled AUC was 0.82 (95% CI, 0.74–0.90). Within the SVM group, Wang et al. (16) showed a relatively wide CI (0.75–0.77), whereas Abujaber et al. (1), Nishi et al. (4), Park et al. (14), and Abedi et al. (17) displayed narrower CI, demonstrating greater stability of the estimate. A random-effect model was applied. However, heterogeneity remained extremely high (*I*^2^ = 100%; *H*^2^ = 26154.71). These findings are illustrated in Figure 11.

**Figure 12:**
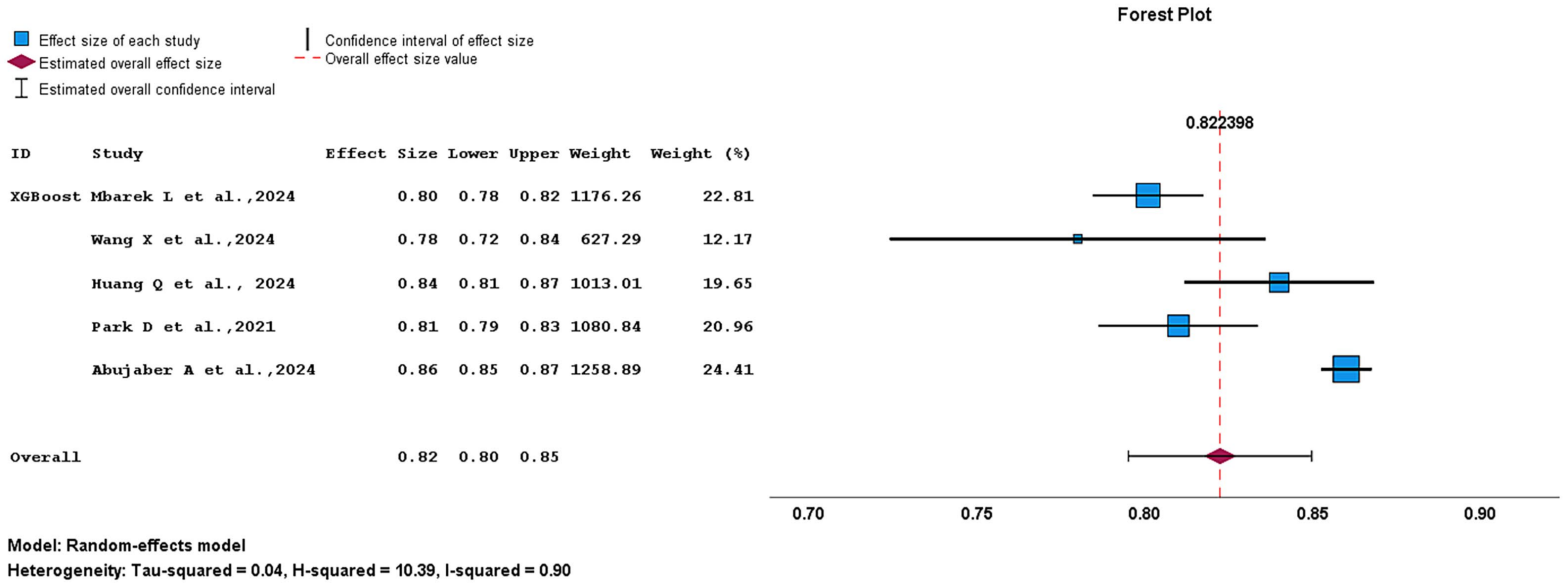
Forest plot summarizing the predictive performance of XGBoost models for Acute Ischemic stroke outcomes.

Figure 12 presents the pooled AUC for XGBoost-based models as 0.82 (95% CI, 0.80–0.85) indicating good predictive performance. Substantial heterogeneity was observed (*I*^2^ = 90%; *H*^2^ = 10.39), suggesting considerable variability across studies. Among the included studies, Wang et al. (16) exhibited the widest CI (0.72–0.84), reflecting lower precision, whereas Abujaber et al. (1) and Huamg et al. (7) showed narrower intervals (0.85–0.87 and 0.81–0.87), indicating greater stability. Abujaber et al. (1) contributed the highest weight (24.41%), while Wang et al. (16) contributed the least (12.17%). Overall, the pooled AUC estimate supports the robustness of XGBoost models for outcome prediction.

**Figure 13:**
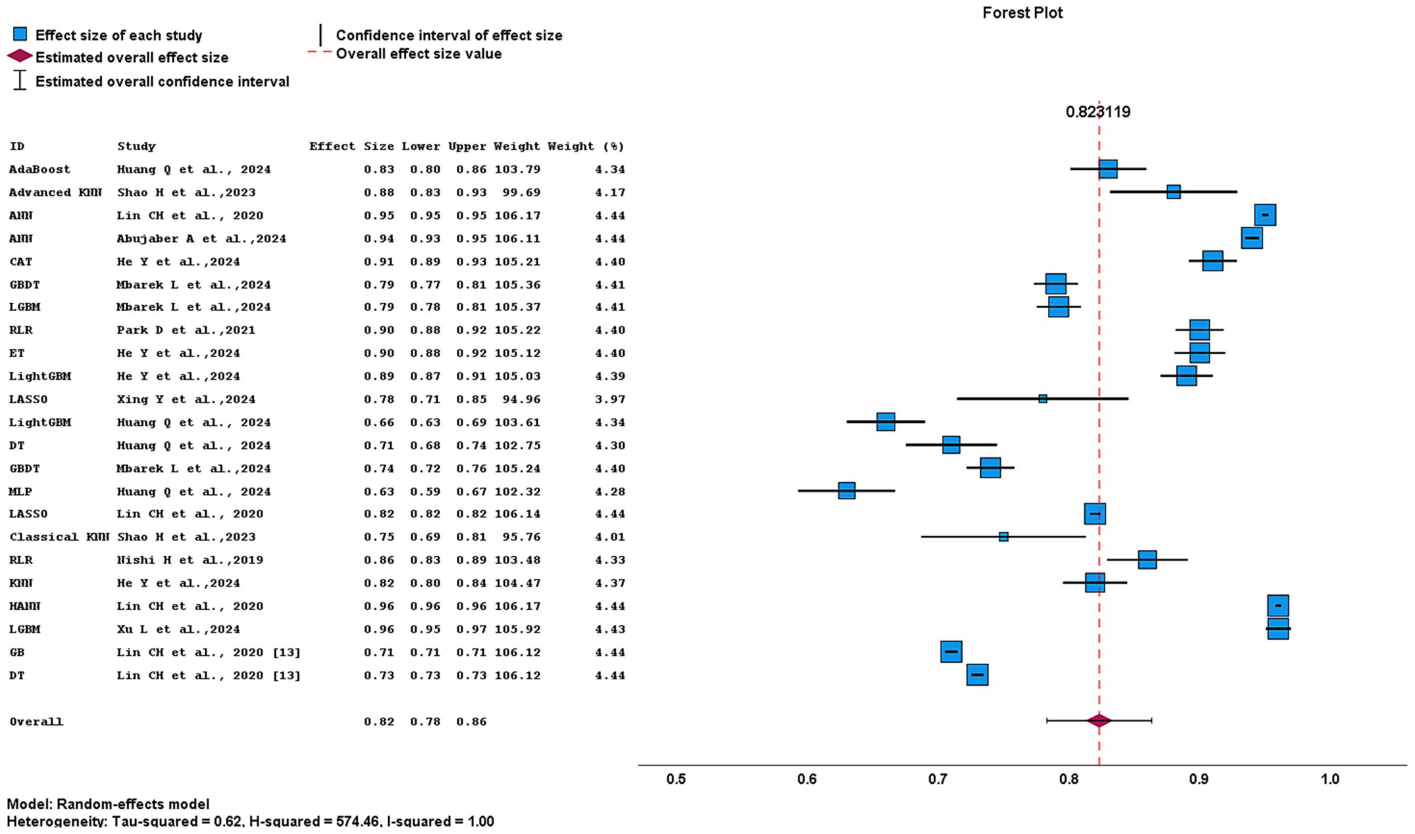
Forest plot showing AUCs of individual ML algorithms across included studies.

Figure 13 presents a pooled AUC of 0.82 (95% CI, 0.78–0.86) across multiple machine-learning algorithms, indicating overall stable predictive performance. Heterogeneity was extremely high (*I*^2^ = 100%; *H*^2^ = 574.46), reflecting substantial variability across studies. Among individual studies, Xing et al. (9) showed the widest CIs, suggesting lower precision, while Abujaber et al. (1), Xu et al. (10), and Lin et al. (13) had narrow CIs, indicating greater stability. Shao et al. (15) contributed minimal weight, as represented by smaller square size. In terms of algorithm performance, tree-based and ensemble algorithms, including LightGBM, and GBDT showed consistent and precise predictive performance, as reflected by their relatively narrow CIs.

Table 5 presents the subgroup analysis indicating that publication year, study design, and algorithm type had a significant impact on heterogeneity, as assessed by chi-square (*Q* statistics), all of which were statistically significant (*p* < 0.001). Studies published in 2021 (*Q* = 7.233) showed relatively lower heterogeneity compared with those published in 2024 (*Q* = 356.476), demonstrated relatively more consistent performance, reflecting gradual methodological refinement in recent research. Study design appeared to be a major moderator, as retrospective cohort studies (*Q* = 1185.596), showed the highest predictive strength but also the greatest variability, compared with retrospective observational studies (*Q* = 12.573). Among algorithms, tree-based models (*Q* = 67.523) showed greater heterogeneity than linear-based models (*Q* = 7.829). Overall, significant heterogeneity was observed across all subgroups, with study design particularly retrospective cohort studies emerging as the most influential factor, followed by publication year and algorithm type. However, subgroup analysis for certain variables could not be performed due to the presence of single studies within those categories.

**Table 5:** Evaluation of heterogeneity across key variables using subgroup analysis.

Variables	*N*	Chi-square (*Q* statistic)	*p*-value
Year			
2021	2	7.233	<0.001
2024	7	356.476	<0.001
Study design			
Retrospective cohort study	5	1185.596	<0.001
Retrospective observational study	3	12.573	<0.001
Algorithms			
Linear based models	3	7.829	<0.001
Tree based models	6	67.523	<0.001

## 4 Discussion

By combining evidence from 12 studies, this meta-analysis evaluated the effectiveness of ML algorithms in predicting outcomes among AIS patients. The pooled AUC across best-performing ML models was 0.87 (95% CI, 0.83–0.91), indicating strong overall discriminative ability and superior predictive performance compared with conventional statistical approaches. Although substantial heterogeneity was observed (*I*^2^ > 99%), the pooled estimate suggests that ML models consistently capture complex determinants of stroke outcomes. The analysis revealed that studies with smaller sample sizes tended to report higher performance estimates, highlighting the importance of large, well-validated datasets for reliable and generalizable prediction.

Across individual algorithms, support vector machine (AUC = 0.85), RF (AUC = 0.85), and XGBoost (AUC = 0.82) consistently outperformed LR (AUC = 0.76). Ensemble approaches, including GBDT and LightGBM, also demonstrated stable and reproducible performance (pooled AUC = 0.83; 95% CI: 0.80–0.86), highlighting the influence of model selection on predictive accuracy. Subgroup analysis further identified study design as the primary source of heterogeneity, with retrospective cohort studies contributing the highest *Q*-value (1185.596), indicating that methodological rigor and dataset quality substantially affect reported performance.

The present findings are consistent with prior AIS-related ML studies evaluating short-term functional outcomes. One study (7) developed nine ML models for 3-month outcome prediction and reported superior performance for RF (AUC = 0.852), closely matching the pooled RF estimate observed in this meta-analysis. Similarly, XGBoost outperformed LR in predicting short-term outcomes among younger AIS patients (11), with NIHSS and baseline mRS identified as key predictors. In another investigation, multiple ML classifiers achieved AUC values exceeding 0.80, with Regularized LR demonstrating performance comparable to established clinical scores such as ASTRAL and ISCORE while improving discrimination through additional predictors (9).

Models developed using larger datasets demonstrated superior predictive performance. A discharge outcome prediction study using nine ML algorithms reported excellent discrimination for RF (AUC = 0.903), with SHAP-based explainability enhancing transparency and clinical applicability (5). Likewise, ANN models trained on the Taiwan Stroke Registry (≈40,293 patients) achieved AUC values approaching 0.95, emphasizing the role of large, high-quality datasets in improving prediction accuracy (13). In contrast, performance declined for long-term recurrence prediction, with AUROC decreasing from 0.79 at 1 year to 0.69 at 5 years (17), suggesting reduced reliability with extended follow-up.

However, substantial heterogeneity observed in this meta-analysis appears to be largely driven by methodological diversity across the included studies. Differences in feature selection, data preprocessing, and validation strategies were frequently noted during data extraction. Several studies excluded cases with incomplete records during preprocessing (1, 4, 7, 9, 11, 14), whereas others applied predefined threshold-based inclusion criteria (5, 15, 17). In contrast, some studies did not clearly report their approach to handling missing data (8, 10, 12, 13, 16). Additional variability arises from differences in hyperparameter optimization, calibration assessment, and the use of internal or cross-validation techniques, which may have influenced the comparability of reported model performance.

Overall, the findings demonstrate that ML models offer strong predictive performance for AIS outcomes, with ensemble-based approaches such as RF, XGBoost, gradient boosting, and LightGBM consistently outperforming traditional linear models. SVM also showed stable performance across multiple datasets. These consistent patterns across algorithms reinforce the suitability of ML for capturing complex predictor–outcome relationships, while explainability methods such as SHAP further support their clinical applicability.

This study has several strengths and limitations. It provides a comprehensive synthesis of recent evidence on ML-based prediction of AIS outcomes using pooled performance estimates and subgroup analyses. However, the search strategy was limited to three databases and was not expanded further. We acknowledge that the exclusion of EMBASE, grey literature, and non-English studies may introduce selection bias. Although the search period began in 2010, all eligible studies were published after 2019, reflecting the emerging nature of this field. Most included studies were retrospective and single-centre, with limited external validation, increasing the risk of overfitting and restricting generalizability. Methodological heterogeneity related to feature selection, missing data handling, calibration assessment, and validation strategies further limited direct comparison of clinical reliability across models. Additionally, incomplete reporting of performance metrics in some studies constrained quantitative synthesis, although these studies were retained due to their methodological relevance.

As the findings suggest, that ML models trained on large, diverse, and high-quality datasets can provide accurate and clinically meaningful predictions to support early decision-making in AIS. To strengthen their reliability and real-world applicability, future studies should prioritize prospective multicenter validation and the integration of multimodal clinical data. Such efforts are critical for improving transparency, reproducibility, and facilitating the successful translation of ML-based prediction models into routine clinical practice.

## 5 Conclusion

This systematic review and meta-analysis demonstrate that ML techniques have strong prognostic potential for predicting outcomes in AIS. Overall, ML-based models achieved high accuracy and performed better than traditional statistical methods, emphasizing their importance in stroke prognosis and management. The findings highlight the effectiveness of ML for early identification of risk and for delivering individualized patient care. In the future, ML can be applied to develop real-time clinical decision-support systems that assist clinicians in identifying high-risk patients, personalizing treatment strategies, and optimizing resource allocation. Finally, by integrating these AI-driven automated systems into routine clinical workflows, healthcare providers can transform stroke management from reactive emergency care to proactive, precision-based interventions that reduce mortality, minimize disability, and improve functional outcomes.

## Data Availability

The original contributions generated for this study are included in the article. Further inquiries may be addressed to the corresponding author.
