# Supplementary material for: Predictive performance of machine learning models in acute ischemic stroke: a systematic review and meta-analysis
Source: Front Neurol. 2026 Mar 11;17:1771341. doi: 10.3389/fneur.2026.1771341 (PMC13012901; doi:10.3389/fneur.2026.1771341)
Supplement: Supplementary file 1 [file Data_Sheet_1.pdf]

Supplementary Table 1: Risk of bias and applicability assessment of included studies using the PROBAST framework.

**Domain 1: Participants**					
**Signalling question**	**Yes**	**Probably Yes**	**Probably No**	**No**	**No Information**
Were appropriate data sources used?	☐	☑	☐	☐	☐
Were inclusion and exclusion criteria appropriate?	☐	☑	☐	☐	☐
Were participants representative of the target population?	☐	☑	☐	☐	☐
**Risk of bias judgment (Participants):**	☑Low	☐ High	☐ Unclear		
**Domain 2: Predictors**					
**Signalling question**	**Yes**	**Probably Yes**	**Probably No**	**No**	**No Information**
Were predictors defined and assessed similarly for all participants?	☐	☑	☐	☐	☐
Were predictors available at the time of outcome prediction?	☐	☑	☐	☐	☐
Were predictor assessments blinded to outcome data?	☐	☐	☑	☐	☐
**Risk of bias judgment (Predictors):** ☑ Low ☐ High ☐ Unclear					
**Domain 3: Outcome**					
**Signalling question**	**Yes**	**Probably Yes**	**Probably No**	**No**	**No Information**
Was the outcome clearly defined?	☐	☑	☐	☐	☐
Was the outcome determined appropriately?	☐	☑	☐	☐	☐
Was outcome assessment blinded to predictor information?	☐	☐	☑	☐	☐
**Risk of bias judgment (Outcome):** ☑ **Low** ☐ High ☐ Unclear					

Domain 4: Analysis					
**Signalling question**	**Yes**	**Probably Yes**	**Probably No**	**No**	**No Information**
Was the sample size adequate?	☐	☐	☑	☐	☐
Were continuous and categorical predictors handled appropriately?	☐	☑	☐	☐	☐
Were missing data handled appropriately?	☐	☐	☑	☐	☐
Was overfitting addressed (e.g., validation, regularization)?	☐	☐	☑	☐	☐
Was model performance evaluated appropriately?	☐	☑	☐	☐	☐
**Risk of bias judgment (Analysis):** ☐ Low ☑ High ☐ Unclear					
**Overall Risk of Bias**					
☐ Low Risk ☑ High Risk ☐ Unclear Risk					
**Applicability Concerns**					
**Domain**	**Low**	**High**	**Unclear**		
Participants	☑	☐	☐		
Predictors	☑	☐	☐		
Outcome	☑	☐	☐		

Supplementary Table 2. Reporting adherence of included prediction model studies across key methodological and performance domains.

Study (Author, Year)	Title / Abstract	Study design & data source	Participants	Outcome defined	Predictors	Sample size	Missing data	Model development	Validation	Performance (AUC)	Calibr ation	Code availa bility	Overall adhere nce (%)

Abujaber A et al., 2024 [1]	Yes	Yes	Yes	No	Yes	Yes	No	Yes	Yes	Yes	Yes	No	**75**
Nishi H et al., 2019 [4]	Yes	Yes	Yes	Yes	Yes	Yes	No	Yes	Yes	Yes	No	No	**75**
He Y et al., 2024 [5]	Yes	Yes	Yes	Yes	Yes	Yes	No	Yes	Yes	Yes	No	No	**75**
Huang Q et al., 2024 [7]	Yes	Yes	Yes	Yes	Yes	Yes	No	Yes	Yes	Yes	Yes	No	**83**
Brugnara G et al., 2020 [8]	Yes	Yes	Yes	No	Yes	Yes	No	Yes	Yes	No	No	No	**58**
Xing Y et al., 2024 [9]	Yes	Yes	Yes	Yes	Yes	Yes	No	Yes	Yes	Yes	Yes	No	**83**
Xu L et al., 2024 [10]	Yes	Yes	Yes	No	Yes	Yes	No	Yes	Yes	Yes	No	No	**67**
Mbarek L et al., 2024 [11]	Yes	Yes	Yes	Yes	Yes	Yes	No	Yes	Yes	Yes	Yes	No	**83**
Bamodu OA et al., 2024 [12]	Yes	Yes	Yes	Yes	Yes	Yes	No	Yes	Yes	No	No	No	**67**
Lin CH et al.,	Yes	Yes	Yes	Yes	Yes	Yes	No	Yes	Yes	Yes	No	No	**75**

2020 [13]													
Park D et al., 2021 [14]	Yes	Yes	Yes	Yes	Yes	Yes	No	Yes	Yes	Yes	Yes	No	**83**
Shao H et al., 2023 [15]	Yes	Yes	Yes	Yes	Yes	Yes	No	Yes	Yes	Yes	No	No	**75**
Wang X et al., 2024 [16]	Yes	Yes	Yes	Yes	Yes	Yes	No	Yes	Yes	Yes	Yes	No	**83**
Abedi V et al., 2021 [17]	Yes	Yes	Yes	No	Yes	Yes	No	Yes	Yes	Yes	No	No	**67**
